# Comparison of Quantitative and Qualitative Tests for Glucose-6-Phosphate Dehydrogenase Deficiency

**DOI:** 10.4269/ajtmh.14-0194

**Published:** 2014-10-01

**Authors:** Nicole LaRue, Maria Kahn, Marjorie Murray, Brandon T. Leader, Pooja Bansil, Sarah McGray, Michael Kalnoky, Hao Zhang, Huiqiang Huang, Hui Jiang, Gonzalo J. Domingo

**Affiliations:** Diagnostics, PATH, Seattle, Washington; Tsuga Analytics, Seattle, Washington; Genomics, Beijing Genome Institute-Shenzhen, Shenzhen, China

## Abstract

A barrier to eliminating *Plasmodium vivax* malaria is inadequate treatment of infected patients. 8-Aminoquinoline–based drugs clear the parasite; however, people with glucose-6-phosphate dehydrogenase (G6PD) deficiency are at risk for hemolysis from these drugs. Understanding the performance of G6PD deficiency tests is critical for patient safety. Two quantitative assays and two qualitative tests were evaluated. The comparison of quantitative assays gave a Pearson correlation coefficient of 0.7585 with significant difference in mean G6PD activity, highlighting the need to adhere to a single reference assay. Both qualitative tests had high sensitivity and negative predictive value at a cutoff G6PD value of 40% of normal activity if interpreted conservatively and performed under laboratory conditions. The performance of both tests dropped at a cutoff level of 45%. Cytochemical staining of specimens confirmed that heterozygous females with > 50% G6PD-deficient cells can seem normal by phenotypic tests.

## Introduction

Glucose-6-phosphate dehydrogenase (G6PD) is a housekeeping enzyme that protects erythrocytes against oxidative injury by providing reducing power in the form of nicotinamide adenine dinucleotide phosphate (NADPH). Erythrocytes are particularly susceptible to oxidative stress, because unlike most cells, they lack other NADPH-producing enzymes.^1^ Rapid destruction of large numbers of erythrocytes in individuals with G6PD deficiency can occur after therapy with certain drugs, including the 8-aminoquinolines used in treating malaria. These episodes can range from mild to life-threatening depending on the dose of the drug, the variant of G6PD deficiency, age (severe reactions are more life-threatening in children), and pre-existing or coexisting morbidities. In the context of malaria treatment, people with G6PD deficiency may experience serious hemolytic episodes if treated with 8-aminoquinolines, such as primaquine; thus, testing for G6PD deficiency before drug administration is essential for patient safety.^2^,^3^

G6PD deficiency affects nearly 400 million people worldwide and is especially prevalent in malaria-endemic areas.^4^–^6^ The *g6pd* gene is located on the X chromosome; thus, females can be homozygous or heterozygous, but males can only be hemizygous for the gene. As a consequence and through lyonization (inactivation of one X chromosome), heterozygous women have two red blood cell populations, each resulting from the expression of one of two G6PD alleles: one population may have normal or deficient G6PD levels, whereas the other population may have another level of deficiency.^7^–^9^ G6PD variants are classified according to the severity of the G6PD deficiency based on the level of enzyme activity compared with normal activity in the population under consideration.^10^ Class I variants cause congenital non-spherocytic hemolytic anemia (< 10% of normal activity). Class II variants cause severe enzyme deficiency (< 10% of normal activity). Class III variants cause moderate to mild enzyme deficiency (10–60% of normal activity). Class IV variants cause very mild or no enzyme deficiency (60–100% of normal activity).

G6PD status is usually determined by measuring enzyme activity in lysate from whole red blood cells with either quantitative or qualitative assays.^11^ However, assays using whole-cell lysate may classify women who are heterozygous for G6PD as normal, even if they have a significant portion of cells that are G6PD-deficient.^12^–^15^ Such cases may present safety considerations. The only way to accurately identify females that are heterozygous for G6PD is by either genotyping or cytochemical staining for intracellular G6PD activity. Cytochemical staining of intracellular G6PD activity allows visualization (by microscopy) or enumeration (by flow cytometry) of the two distinct red cell populations resulting from the G6PD-normal and -deficient allele expression.^16^–^18^ Quantitative assays do allow the discrimination of intermediate to normal levels with fine resolution; however, tests of this type currently on the market require advanced laboratory infrastructure and skilled personnel. A commonly used qualitative assay is the fluorescent spot test (FST), which can be performed in some low-resource areas and can identify severe deficiencies. However, current commercially available tests require advanced infrastructure and skilled personnel, which limit their use out of laboratory settings. Of the qualitative G6PD tests, the FST is most commonly performed, including in some low-resource areas. Although the FST is able to identify severe deficiencies, discrimination of intermediate levels with this test is more difficult. Developing a robust, quantitative point-of-care G6PD test for field use in low-resource areas is a high priority for overall malaria control and elimination.^2^,^3^

The purpose of this study was to perform a highly controlled and standardized performance comparison of several commercially available G6PD tests. This study assessed the accuracy of each test in the identification of various levels of G6PD deficiency under the same operating conditions with the same blood samples. Data are presented describing (1) the correlation between two quantitative tests, (2) the performance of two qualitative tests against the selected reference quantitative test, and (3) the relationship between intracellular G6PD activity level assayed by a cytochemical staining method and the quantitative G6PD status by the reference test.

## Materials and Methods

### Subjects and sample collection.

All blood samples were obtained from Bioreclamation, Inc. (Westbury, NY) and collected between September of 2012 and July of 2013 from volunteers who were at least 18 years of age and signed consent under Institutional Review Board Protocol 2010-017. All volunteers were of African-American origin. Specimens were transported in ethylenediaminetetraacetic acid (EDTA) anticoagulant venipuncture vacuum tubes on cold packs and stored at 4°C. Specimen processing took place between 2 and 4 days after blood collection. All enzyme activity assays were conducted on the same day for each blood sample, and cytochemical staining assays were conducted within 24 hours. Blood lysis always was performed immediately before conducting an enzyme assay as part of the test protocol. No personal identification data were collected, and all G6PD assays were performed independently and blinded to G6PD status.

### Trinity Biotech quantitative G6PD test (reference assay).

All specimens were characterized for G6PD activity in duplicate with the quantitative G6PD kit from Trinity Biotech (catalog number 345-B; Trinity Biotech PLC, Bray, Ireland) according to the manufacturer's instructions as the reference assay for all testing. Normal, intermediate, and deficient Trinity controls (catalog numbers G6888, G5029, and G5888, respectively) were run using the same method on each day of testing.

Briefly, 10 μL whole blood in anticoagulant was added to 1 mL reagent and incubated at room temperature for 5 minutes. Two milliliters of substrate was added to the solution and mixed by inversion. One milliliter of the mixture was aliquoted into each of two ultraviolet (UV) -transparent disposable cuvettes (catalog number 47727-024; Brand Co., Wertheim, Germany). The duplicate cuvettes were incubated at 30°C in a water bath for 5 minutes. Enzyme activity was determined using a temperature-regulated spectrophotometer (UV-1800 Shimadzu; Shimadzu Scientific Instruments, Columbia, MD) set at 30°C by measuring the change in rate in absorbance at 340 nm over 5 minutes. G6PD activity values were calculated in units per gram hemoglobin (Hb). Hb concentration was determined using a Hemocue Hemoglobin System (HemoCue Hb 201 + Analyzer, no. 121721, catalog number 22-601-007; Fisher Scientific, Inc., Waltham, MA).

### R&D Diagnostics Ltd. quantitative test.

G6PD activity was measured in units per gram Hb with the R&D Diagnostics Ltd. (Athens, Greece) quantitative enzymatic colorimetric method (catalog number ODMMR2000-D) according to the manufacturer's instructions. Briefly, reagent mixture was reconstituted and pre-warmed to 30°C. Five microliters whole blood with anticoagulant from each subject was added in duplicate to wells in a flat-bottom 96-well plate. Normal, intermediate, and deficient controls were run with each plate (catalog numbers G6888, G5029, and G5888, respectively; Trinity Biotech). Seventy-five microliters elution agent was added to each well and mixed by pipet three times. The plate was incubated at 30°C for 10 minutes. Fifteen microliters eluant of each sample was transferred to a new round-bottom 96-well plate, and 75 μL reagent mixture and 80 μL color reagent/booster mixture (mixed 1 part booster with 10 parts reagent) were added and mixed by pipet three times. The plate was equilibrated at 30°C for 3 minutes and then read in a plate reader set to 30°C. Results were read at 60-second intervals for 20 minutes at 550 nm. After the 20-minute reading, the plate was read again for a single time point at 405 nm. G6PD enzyme activity was calculated by determining change in absorbance at 550 nm over 12 minutes, multiplying by a dilution factor, and dividing by the absorbance reading at 405 nm.

### Trinity Biotech fluorescent spot test.

Each specimen and the Trinity normal, intermediate, and deficient controls were tested using Whatman No. 1 filter paper (catalog number 1001-150; GE Healthcare UK Limited, Buckinghamshire, UK) with the Trinity Qualitative G6PD FST Kit (catalog number 203-A; Trinity Biotech) according to the manufacturer's instructions. The method detects the fluorescence of NADPH, which is proportional to G6PD activity, under long-wave UV light (365 nm). Controls (Trinity normal, intermediate, and deficient as described previously) were tested the same day as specimens. Briefly, 10 μL blood was added to 200 μL reagent mixture and spotted onto filter paper at time 0. Each sample was incubated at 37°C and spotted again after 5 and 10 minutes of incubation. Fluorescence was observed for the three time points after samples had dried. Fluorescence intensity was used to classify specimens into three groups of enzyme activity: normal (moderate to strong fluorescence after 5 minutes and strong fluorescence after 10 minutes), intermediate (weak fluorescence after 5 minutes and weak to moderate fluorescence after 10 minutes), and deficient (very faint or no fluorescence after 10 minutes).

### BinaxNOW qualitative test.

Blood specimens were evaluated on the lateral-flow colorimetric test platform BinaxNOW G6PD Test (catalog number 780-000; Alere Inc., Waltham, MA), which must be performed between 18°C and 25°C. Tests were performed according to the manufacturer's instructions at a mean temperature of 20.2°C (range of 18°C to 23°C). G6PD Trinity controls (normal and deficient only) were assessed periodically to ensure quality performance of the BinaxNOW Test.

### Cytochemical staining and flow cytometry.

Whole-blood specimens were characterized for intracellular G6PD activity by flow cytometry as described previously.^16^ Briefly, 10 μL 50% hematocrit red blood cell suspension was diluted into 90 μL 0.9% NaCl, combined with 100 μL sodium nitrite (0.125 M; Sigma-Aldrich, St. Louis, MO), and incubated at room temperature for 20 minutes. Samples were washed three times with phosphate-buffered saline (PBS), centrifuged at 3,000 rpm for 3 minutes, and resuspended in 100 μL PBS. The red blood cells were then combined with 18 μL glucose (0.28 M) in PBS and 6 μL Nile Blue Sulphate (0.01%; Sigma-Aldrich) and incubated at 37°C for 90 minutes in open Eppendorf tubes (Hamburg, Germany). After incubation, 2.5 μL 0.4 M potassium cyanide (Sigma-Aldrich) was added and incubated for 5 minutes; 5 μL each sample was added to 100 μL 3% hydrogen peroxide in PBS, agitated vigorously by hand, and washed two times in PBS. Specimens were analyzed using a FACScaliber cytometer (BD Biosciences, San Jose, CA) (10,000 events) in the FL1 channel (533 ± 30 nm). The percent normal and deficient cells were calculated as described previously.^16^ Briefly, maxima in the kernel density estimation function were identified, and the midpoint between the two maxima was used as an arbitrary gate between high- and low-intensity subpopulations. When only one maximum was found (for example, for G6PD-deficient homozygotes), the gate was placed 0.5 log units away.

### DNA extraction.

Peripheral blood mononuclear cells (PBMCs) were separated from 1 mL whole blood using Lymphoprep (catalog number 1114547; Axis-Shield, Oslo, Norway) and resuspended in 1 mL PBS. DNA was extracted from PBMCs using the Qiagen Blood and Cell Culture DNA Kit 20/G (catalog number 13323; Valencia, CA). The DNA extraction was performed according to the manufacturer's instructions. The DNA pellet was resuspended in 100 μL 10 mM Tris-HCl (pH 8.0) and incubated at 55°C for 1–2 hours. DNA samples were stored at −20°C.

### DNA sequencing.

The DNA libraries from genomic DNA (gDNA) of proband and controls were constructed according to Illumina paired-end libraries construction protocol. Briefly, an ultrasonoscope (Covaris S2, Woburn, MA) was used to fragment the gDNA into fragments of 200–300 bp. Purified DNA were treated with T4 DNA polymerase, T4 phosphonucleotide kinase, and the Klenow fragment of *Escherichia coli* DNA polymerase to fill 5′ overhangs and remove 3′ overhangs. Single nucleotide of A is added to the terminal of 3′ ends of blunt fragments. Adapter oligonucleotides from Illumina were ligated to the ends, and a four-cycle polymerase chain reaction (PCR) reaction was performed after adapter ligation.

A custom design array, which contains all the exon sequences and their flanking sequences of the G6PD, was used in this study; after hybridization and washing, sequencing was then performed with the HiSeq2000 (Illumina, San Diego, CA) to produce paired-end reads (approximately 90 bp at each end) according to the manufacturer's standard cluster generation and sequencing protocol. Image analysis and base calling were performed using the Illumina Pipeline (version 1.3.4) to generate primary data. Indexed primers were used to identify the different reads from different samples in the primary data. The reads that could not be perfectly matched to theoretical adapter indexed sequence were filtered followed by the removal of low-quality reads from the primary data using a local dynamic programming algorithm, and the remaining reads were considered suitable for additional analysis. The clean reads with a length of 90 bp were then subjected to alignment against NCBI37/hg19 assembly of the human genome^19^ using the Burrows Wheeler Aligner (BWA) software. Single nucleotide polymorphisms (SNPs) and indels were called using the Genome Analysis Toolkit (GATK) (Broad Institute, Cambridge, MA).

### Statistical methods.

All statistical analyses were conducted in Stata 12.0 (Statacorp, College Station, TX). The Trinity quantitative test was used as the reference assay. The mean, SD, median, and range for G6PD activity were estimated for the entire study population and by sex. To evaluate the performance of other G6PD tests, the cutoff points for levels of G6PD deficiency were determined as previously described.^2^ Briefly, the adjusted G6PD median activity value for normal males was calculated (excluding males with severe G6PD deficiency defined as ≤ 10% of the G6PD median value for all males in the study population), and different cutoff levels of activity were defined (i.e., 10%, 20%, 30%, and 60% of the adjusted male median value). Individuals were classified as G6PD-deficient (less than or equal to the cutoff value) or normal (more than the cutoff value) based on these levels. The sensitivities, specificities, positive predictive values (PPVs), and negative predictive values (NPVs) of the BinaxNOW and the Trinity FST were evaluated for each of the cutoff points.

The number and percentage of patients classified into each test category for the BinaxNOW and Trinity FST were calculated (normal and deficient for both and intermediate for the FST); for additional assessment of the FST, normal and intermediate results were combined for one analysis, and deficient and intermediate were combined for another analysis. To evaluate the relationship between the Trinity quantitative and R&D quantitative tests, we calculated the Pearson's correlation coefficient.

## Results

### Study population.

Blood samples were collected from 214 healthy African-American subjects ages 19–59 years old; one-half were male, and one-half were female (107 each). All tests for G6PD activity were performed blinded to the patient's G6PD status.

### Quantitative determination of G6PD activity.

**Figure 1.**
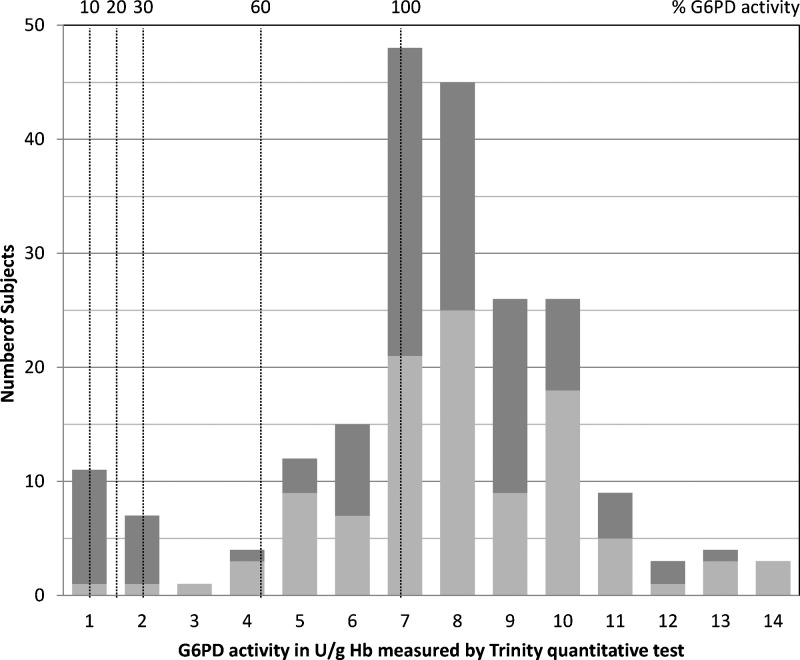
Distribution of G6PD activities for the study population described in Table 1 indicating 10%, 20%, 30%, 60%, and 100% of normal activity for this population. Normal activity was defined as the adjusted G6PD median activity value for normal males. Females are indicated by light gray bars, and males are indicated by dark gray bars. *N* = 214 (107 females and 107 males).

All quantitative results were determined at 30°C. The G6PD enzyme activity reference values in the study population using the Trinity Biotech G6PD quantitative test are given in Table 1. G6PD reference values for this study were determined as previously described.^2^ Six males had activity levels ≤ 10% of the median value for all males in the study population and were excluded for calculation of the adjusted G6PD median activity value for normal males. This adjusted value is used to calculate various cutoff levels (Figure 1

) to avoid having severely deficient values skew the population median. The median G6PD activity for the entire population was 7.30 U/g Hb (range = 0.12–14.04 U/g Hb), and the adjusted male median was 7.18 U/g Hb (range = 0.84–12.26 U/g Hb). Figure 1 shows the distribution of G6PD activities in the study population, with the 100% activity level (7.18 U/g Hb) indicated. Various cutoff levels are also indicated as percentages of adjusted male normal. The G6PD activities for each of the cutoff values—10%, 20%, 30%, and 60% of normal—are given in Table 2.

**Figure 2.**
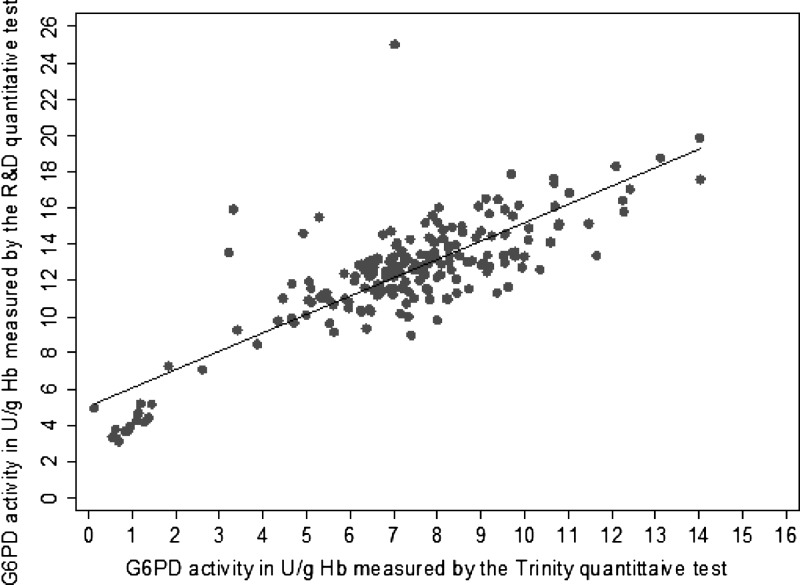
Correlation between G6PD activities measured by Trinity and R&D quantitative tests (*N* = 214). The Pearson correlation coefficient is 0.7585. *N* = 214.

As shown in Figure 2, there was a moderately strong correlation between G6PD activity levels measured by the Trinity and the R&D quantitative tests, with a Pearson correlation coefficient of 0.7585. For the R&D quantitative tests, the median G6PD activity for the entire population was 10.20 U/g Hb (range = 2.12–19.02 U/g Hb), and the adjusted male median was 9.39 U/g Hb (range = 2.12–16.00 U/g Hb). The mean absolute activity value was higher when measured by the R&D test (10.33 U/g Hb) than the Trinity test (7.17 U/g Hb). For all these values, the differences between the two tests were statistically significant (*P* < 0.001). Both tests clustered severe G6PD-deficient samples in the low G6PD activity range.

### Qualitative determination of G6PD activity.

Of 214 subjects in the study, samples from 201 subjects were evaluated with the BinaxNOW G6PD test. Results indicated that 182 (90.5%) subjects had normal G6PD activity levels and that 19 (9.5%) subjects were deficient. The 19 deficient samples identified by BinaxNOW are identical to the 18 samples shown below the 30% cutoff with the reference assay plus the 1 additional sample directly above this cutoff (Figure 1).

The FST was performed on all 214 specimens. Among these specimens, 189 (88.3%) were determined to have normal G6PD activity, 13 (6.1%) were determined to be deficient, and 12 (5.6%) had intermediate levels when interpreted according to the manufacturer's instructions. The range of activity for specimens classified as intermediate by the FST test was 0.12–5.05 U/g Hb, with a mean activity of 2.80 U/g Hb. Importantly, one FST intermediate had G6PD activity below 10% of normal activity, four FST intermediates were below 20% of normal activity, and five FST intermediates were below 30% of normal activity. In a subsequent analysis of the data, intermediate values were counted in two ways—grouped with the deficient specimens or the normal specimens—to assess test performance against the reference quantitative test (Table 2). When intermediates were counted as deficient, 25 (11.7%) subjects were classified as deficient; when intermediates were included with normal subjects, the number deficient was 13 subjects.

Table 2 is a summary of the performance of the two qualitative assays evaluated against the Trinity quantitative test. The BinaxNOW assay had 100% sensitivity and 100% NPV for the 10%, 20%, and 30% activity cutoff values, but sensitivity dropped to 82.6% when the cutoff level was 60% of normal, and NPV fell to 97.8%. The FST results most closely matched the quantitative test results when specimens with intermediate levels were added to those with deficient values, with 100% sensitivity and 100% NPV at the 10%, 20%, and 30% activity cutoff values. The FST was more accurate for measuring sensitivity and NPV (91.3% and 98.9%, respectively) than the BinaxNOW at the 60% cutoff value (Table 2). FST accuracy fell for these parameters when intermediate values were added to normal values.

**Figure 3.**
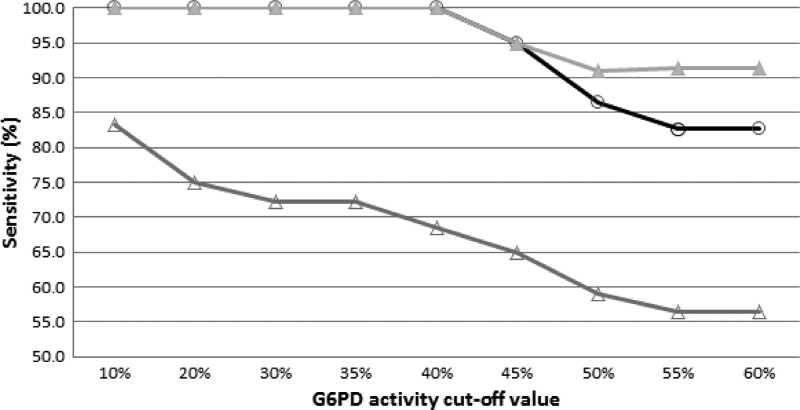
Sensitivity of two qualitative G6PD tests, the BinaxNOW and Trinity FST, at various cutoff levels for enzyme activity between 10% and 60% of the adjusted normal activity. Gray triangles = FST with intermediate samples counted as deficient; white circles = BinaxNOW; white triangles = FST with intermediates counted as normal. *N* = 214.

Additional analysis of the data indicated reduced sensitivity for both the BinaxNOW and FST qualitative tests at the 45% cutoff level (Figure 3).

### Cytochemical staining and flow cytometry.

**Figure 4.**
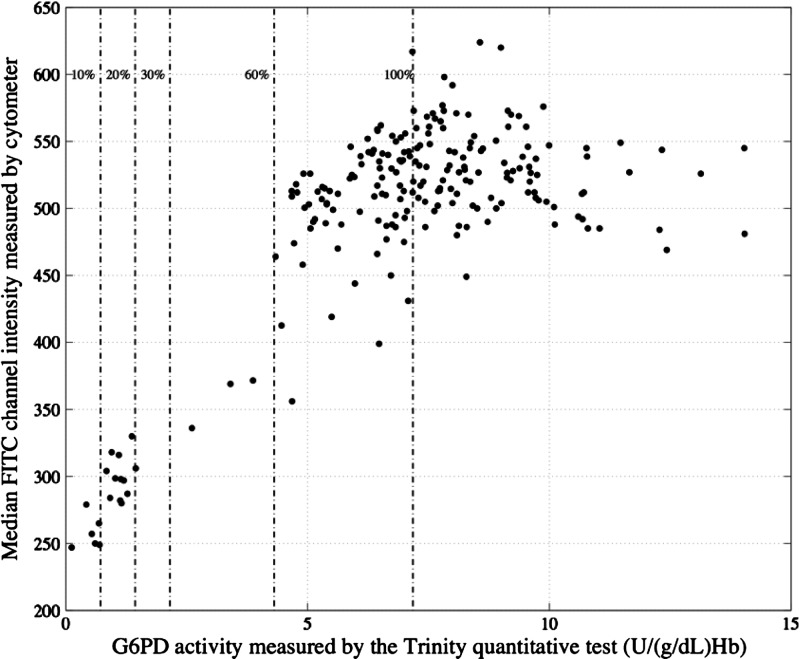
Relationship between median FITC fluorescence of intact red blood cells assayed by a cytochemical staining test and G6PD activity measured in lysed samples from the same subjects by the quantitative Trinity test.

**Figure 5.**
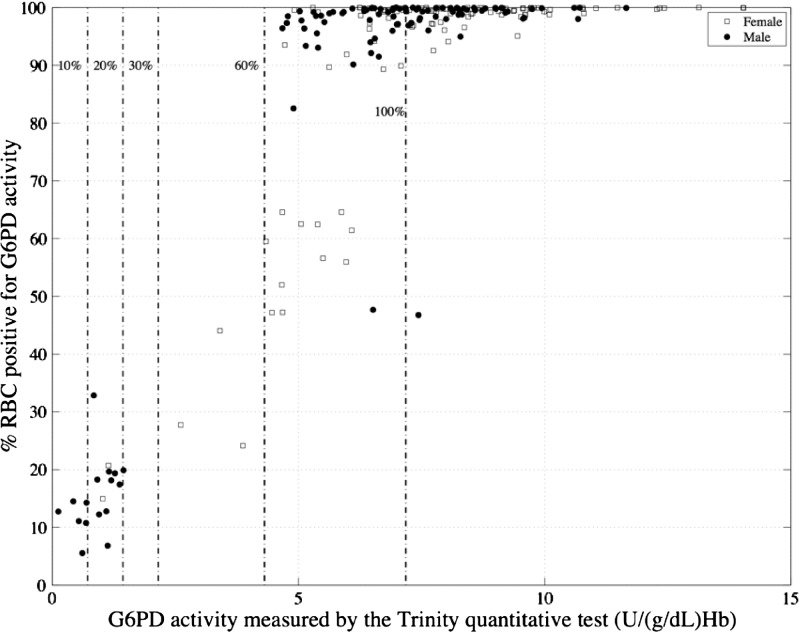
Relationship of G6PD activity measured by the Trinity quantitative test in whole-blood lysate (*x* axis) and percent cells with normal G6PD activity measured by flow cytometry (*y* axis); 10%, 20%, 30%, and 60% cutoff levels as well as 100% normal activity by the Trinity test for the study population are shown. *N* = 214. RBC = red blood cell.

The cytofluorometric assay was performed on all samples with automated measurement in a flow cytometer. Figure 4

shows the correlation between median fluorescein isothiocyanate (FITC) values determined by flow cytometry and G6PD activity determined by the Trinity quantitative reference assay. Using either median FITC or total FITC gave similar correlations (data not shown for total FITC). The flow cytometry data were also analyzed to quantify the percentage of red blood cells with normal G6PD activity as previously described.^16^ Figure 5

shows the correlation between the percentage of cells with normal activity determined by flow cytometry and whole-blood cell lysate G6PD activity as determined by the reference quantitative test.

### DNA sequencing results.

**Figure 6.**
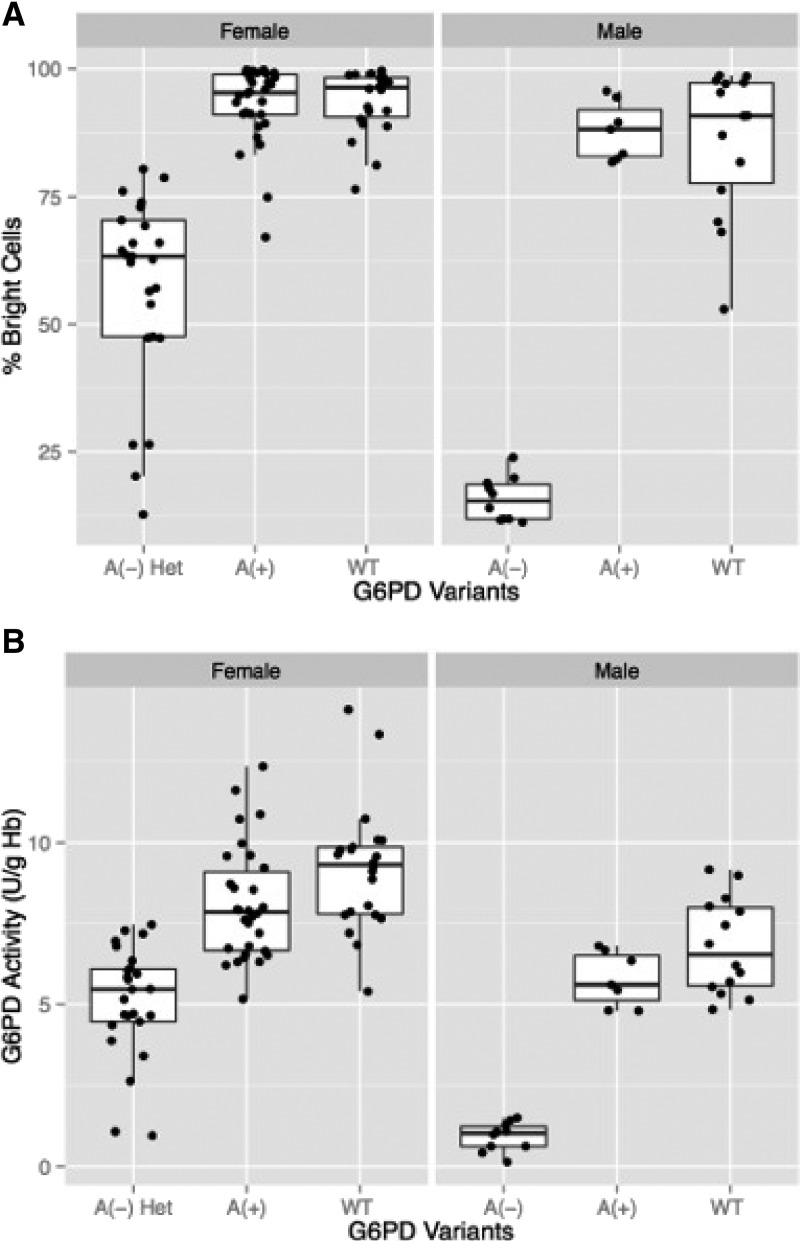
Block charts showing the mean (horizontal lines), 25 and 75 percentiles (white rectangles), and 5 and 95 percentiles (extremities of vertical lines) for (**A**) percent bright cells as determined by flow cytometry and (**B**) G6PD activity for the different genotypes identified in 110 samples sequenced in this study. A(−), A(+), and WT males refers to males hemizygous for A(−), A(+), and normal G6PD alleles. A(−) Het females = all heterozygous females with at least one A− allele; A(+) = all heterozygous females with one normal allele and one A(+) allele as well as two homozygous A (+) females; WT females = females homozygous for the normal G6PD allele.

The G6PD gene was sequenced for 110 of 214 samples, comprising 79 females and 31 males. Selection of specimens for sequencing was biased toward patients with low G6PD activity levels and females. The final set of specimens sequenced included all 6 females with activity < 4.308 U/g Hb (60% cutoff), 34 of 39 females with ≤ 100% of normal activity (6.99 U/g Hb), 39 of 68 females with activity > 100% of normal activity, 4 of 6 males with activity < 10% of normal, 11 of 17 males with < 60% of normal G6PD activity, and the remaining 16 male samples with activity throughout the remaining G6PD activity dynamic range. Table 3 provides a summary of the G6PD genotypes and associated G6PD activities for 110 sequenced genes. In addition, 96 synonymous mutations (changes in DNA sequence with no impact on the protein sequence) were identified (data not shown). Figure 6 shows the percentage of bright cells and the G6PD activities for the different genotypes identified in this subset of study samples. Males hemizygous for A(−) had < 25% bright cells and < 20% of normal G6PD activity. Males hemizygous for A(+) showed results similar to those with the normal G6PD allele. One female was heterozygous for both A−^(202)^ and A−^(968)^ and correspondingly, had both a low percentage of bright cells and low G6PD activity. Females with an A(−) allele and either an A(+) or a normal allele showed the broadest range of G6PD activity and percentage of bright cells, but values were still significantly (*P* < 0.001) below those for females homozygous for normal G6PD activity or heterozygous for normal and A(+).

## Discussion

A challenge in assessing the performance of currently available tests for G6PD deficiency is poor harmonization in evaluation criteria. One example is the problem of defining normal G6PD activity, which typically is described as the mean or median activity for a given population including deficient and heterozygous values. However, the prevalence of G6PD deficiencies, which ranges from < 0.1% to > 15%, can significantly influence the population normal value.^4^ Robust G6PD reference normal values for a given population can only be obtained using large sample numbers, but this is not feasible for evaluation studies of tests for G6PD deficiency. When smaller sample numbers are used, the prevalence of severely deficient individuals may skew the population normal value. In practice, there is wide variability in how G6PD tests are evaluated.^14^,^20^–^23^

A recent report recommended the use of standardized evaluation criteria for G6PD tests to better harmonize the performance comparison studies of different G6PD diagnostic tests.^2^ This approach includes using a quantitative test to measure activity levels and establish a normal G6PD activity reference value for a study population. The standardized normal reference value (adjusted male median) is established for a study population by taking the median G6PD activity value of the male population excluding males with severe deficiency (< 10% of the median activity) to minimize the impact of prevalence of severely deficient individuals on the reference normal value. For this study, a commercially available spectrophotometric quantitative test, the Trinity quantitative assay, was selected as the reference assay to define normal G6PD activity and used for analysis of the samples.^24^,^25^ Using this approach for this study, 6 males were identified as severely deficient, with activity ≤ 10% of the G6PD median value for all 107 males in the study population. These values were removed from the study population, and the resulting adjusted male median was used as the normal value.

A comparison of the reference test with another commercially available quantitative test showed that, although both tests clustered severely deficient specimens, the overall correlation was moderate, and the absolute activity values were significantly different for the same samples between the two tests. Criteria for selecting the cutoff value on one test could not be applied to the other test. The difference in G6PD activity determined by the two assays highlights the sensitivity of enzyme activity to reaction conditions as well as platforms. Thus, the need to harmonize to a reference standard assay should be considered for the evaluation of new tests for G6PD deficiency, including those intended for point-of-care use. A recent study highlights the impact of specimen handling and assay conditions on G6PD test performance.^26^ The effects of different conditions on assay performance are likely to be assay- and platform-dependent.^2^

The G6PD activity as determined by the Trinity quantitative test and the adjusted male median activity were used to evaluate the performances of two qualitative tests, the BinaxNOW G6PD Test and the Trinity FST. Using 10%, 20%, or 30% of the adjusted male normal value as different cutoffs for deficiency, both qualitative tests were 100% accurate when intermediate results were grouped with G6PD-deficient samples for the FST. Of 12 specimens with intermediate FST results, as noted above, 1 specimen had G6PD activity levels below 10% of normal, 4 specimens had G6PD activity levels below 20% of normal, and 5 specimens had G6PD activity levels below 30% of normal. It is not clear if these discordant results are a consequence of poor test performance or a reflection of measuring enzyme activity under different reaction conditions (such as temperature and dilution). Accuracy for both tests fell when the cutoff level was ≥ 45% of normal (Figure 3). Similar results for performance of the BinaxNOW G6PD Test were previously reported.^23^ Discrepancy between the FST and quantitative test results also has been reported previously.^12^,^14^,^15^ In the previously published reports and the results presented here, the tests were run by laboratory experts in highly regulated laboratory settings, conditions that are not likely to be found in low-resource settings. In this study, the qualitative tests were performed at low ambient temperatures (mean temperature of 20.2°C; range = 18°C to 23°C). Given the temperature dependence of enzyme activity and the probable higher operational temperatures in malaria-endemic settings, it is likely that the performance of these tests will decline further at lower cutoff values in those settings.

Cytochemical staining presents an alternative way to assess G6PD deficiency, and because it assays intact red blood cells, it can identify heterozygous females.^16^–^18^ A recently described cytofluorometric method using flow cytometry to measure fluorescence in intact cells was used in our study to compare intracellular G6PD activity distributions with the reference assay G6PD activity in lysed cells for each sample.^16^ Using this latter method, this study indicated that females with as much as 58% of their red blood cell population deficient in G6PD activity (assayed by cytofluorometric method) and cytometry profiles indicative of heterozygotes would be classified by the quantitative assay as above a cutoff value of 60% activity and thus, normal using the analysis algorithm described previously.^16^ In a recent report combining microscopy with cytochemical staining, patients with > 20% of red blood cells deficient in G6PD activity were classified as G6PD-deficient.^14^ In this study, all severely deficient participants (with < 10% G6PD activity) had 85% or more cells deficient in G6PD activity as determined by flow cytometry. Participants with < 30% G6PD activity had 67% or more cells deficient in G6PD activity. These discrepancies most likely arise as a consequence of the differences in platforms (microscopy versus flow cytometry) and analysis algorithms. Additional development of the cytochemical methodologies could provide a powerful tool for analysis of G6PD activity level and susceptibility to oxidative challenges.

G6PD sequencing showed that genotypes corresponded to phenotypes by both G6PD activity and cytochemical staining, with the exception of females heterozygous for an A(−) allele and a normal or A(+) allele, which displayed a broad range of G6PD activities and portion of bright cells. The data presented here confirms that the A(−) G6PD allele can result in severe G6PD deficiency (< 10% normal activity), even if it is currently classified as a class III G6PD variant.

Tests evaluated under this study are inappropriate for use in low-resource settings because of either their functional temperature working range or their complexity. The development of high-quality point-of-care tests for G6PD deficiency that are suitable for use in low-resource areas remains a critical need for supporting treatment strategies in malaria control and elimination efforts. Adhering to harmonized evaluation protocols and using standardized reference assays will be essential for accurately assessing the performance of new diagnostic products in various populations with different G6PD deficiency traits and epidemiology.

## Figures and Tables

**Table 1:** Reference values for G6PD activity in the study population by Trinity quantitative test

Reference values (U/g Hb)	Total (*N* = 214)	Female (*N* = 107)	Male (*N* = 107)	Adjusted male* (*N* = 101)
Mean	7.17	7.70	6.63	6.99
SD	2.67	2.39	2.83	2.48
Median	7.30	7.69	7.06	7.18
Range	0.12–14.04	1.03–14.04	0.12–12.26	0.84–12.26

* These values exclude males with severe G6PD deficiency defined as ≤ 10% of the G6PD median value for all males in the study population.

**Table 2:** Clinical performance of the BinaxNOW test and the Trinity FST for detection of deficiency in G6PD activity compared with Trinity quantitative test.

Cutoff value, U/g Hb (percent of adjusted normal male median value = 7.18)	10% Activity cutoff	20% Activity cutoff	30% Activity cutoff	60% Activity cutoff
Cutoff value, U/g Hb	0.718	1.436	2.154	4.308
No. of samples with G6PD levels below cutoff (%) [no. of M + no. of F]	6 (2.80) [6 M]	16 (7.48) [15 M + 1 F]	18 (8.41) [16 M + 2 F]	23 (10.75) [17 M + 6 F]
BinaxNOW G6PD test				
Sensitivity % (95% CI)	100.0 (54.1–100.0)	100.0 (79.4–100.0)	100.0 (81.5–100.0)	82.6 (61.2–95.0)
Specificity % (95% CI)	93.3 (88.9–96.4)	98.4 (95.3–99.7)	99.5 (97.0–100.0)	100.0 (97.9–100.0)
PPV % (95% CI)	31.6 (12.6–56.6)	84.2 (60.4–96.6)	94.7 (74.0–99.9)	100.0 (82.4–100.0)
NPV % (95% CI)	100.0 (98–100.0)	100.0 (98–100.0)	100.0 (98.0–100.0)	97.8 (94.5–99.4)
Trinity fluorescent spot test*				
Sensitivity % (95% CI)	100.0 (54.1–100.0)	100.0 (79.4–100.0)	100.0 (81.5–100.0)	91.3 (72.0–98.9)
Specificity % (95% CI)	90.9 (86.1–94.4)	95.5 (91.5–97.9)	96.4 (92.8–98.6)	97.9 (94.7–99.4)
PPV % (95% CI)	24.0 (9.36–45.1)	64.0 (42.5–82.0)	72.0 (50.6–87.9)	84.0 (63.9–95.5)
NPV % (95% CI)	100.0 (98.1–100.0)	100.0 (98.1–100.0)	100.0 (98.1–100.0)	98.9 (96.2–99.9)
Trinity fluorescent spot test†				
Sensitivity % (95% CI)	83.3 (35.9–94.6)	75.0 (47.6–92.7)	72.2 (46.5–90.3)	56.5 (34.5–76.8)
Specificity % (95% CI)	96.2 (92.6–98.3)	99.5 (97.2–100.0)	100.0 (98.1–100.0)	100.0 (98.1–100.0)
PPV % (95% CI)	38.5 (13.9–68.4)	92.3 (64.0–99.8)	100.0 (75.3–100.0)	100.0 (75.3–100.0)
NPV % (95% CI)	99.5 (97.3–100.0)	98.0 (95.0–99.5)	97.5 (94.3–99.2)	95.0 (91.0–97.6)

CI = confidence interval; F = female; M = male.

* Trinity fluorescent spot test: intermediate test results combined with deficient test results.

† Trinity fluorescent spot test: intermediate test results combined with normal test results.

**Table 3:** G6PD genotypes determined by sequencing and associated G6PD activity values

Mutation	Zygosity	Amino acid substitutions	*N*	G6PD values (U/g Hb)		
				Range	Median	Mean (SD)
Male						
Normal	Hemizygous	−	14	4.9–9.14	6.54	6.8 (1.44)
A(+)	Hemizygous	N126D	7	5.1–6.67	6.21	5.98 (0.59)
A−^(202)^	Hemizygous	N126D and V68M	10	0.12–1.45	1.03	0.93 (0.41)
Female						
Normal	Homozygous	−	23	5.4–14.04	9.45	9.17 (1.88)
A+	Homozygous	N126D	2	6.44–7.25	N/A	6.85 (0.57)
A+	Heterozygous	N126D	28	5.29–12.33	7.81	8.06 (1.75)
A−^(202)^	Heterozygous	N126D and V68M	23	1.84–7.33	5.05	5.22 (1.47)
A−^(968)^	Heterozygous	N126D and L323P	1	N/A	5.5	N/A
A−^(202)^ and A−^(968)^	Heterozygous	N126D, V68M, and L323P	1	N/A	1.03	N/A
Ilesha	Heterozygous	E156K	1	N/A	7.12	N/A

Synonymous mutations are not included. Sequencing was performed on 110 individual samples. N/A = not applicable.

## References

[1] Beutler E (1994). G6PD deficiency. Blood.

[2] Domingo GJ, Winasti Satyagraha A, Anvikar A, Baird JK, Bancone G, Bansil P, Carter N, Cheng Q, Culpepper J, Eziefula C, Fukuda M, Green J, Hwang J, Lacerda M, McGray S, Menard D, Nosten F, Nuchprayoon I, Nwe Oo N, Bualombai P, Pumpradit W, Qian K, Recht J, Roca A, Satimai W, Sovannaroth S, Vestergaard LS, von Seidlein L (2013). G6PD testing in support of treatment and elimination of malaria: recommendations for evaluation of G6PD tests. Malar J.

[3] von Seidlein L, Auburn S, Espino F, Shanks D, Cheng J, McCarthy J, Baird JK, Moyes C, Howes R, Menard D, Bancone G, Winasti-Satyahraha A, Vestergaard LS, Green J, Domingo GJ, Yeung S, Price R (2013). Review of key knowledge gaps in glucose-6-phosphate dehydrogenase deficiency detection with regard to the safe clinical deployment of 8-aminoquinoline treatment regimens: a workshop report. Malar J.

[4] Howes RE, Piel FB, Patil AP, Nyangiri OA, Gething PW, Dewi M, Hogg MM, Battle KE, Padilla CD, Kaird JK, Hay SI (2012). G6PD deficiency prevalence and estimates of affected populations in malaria endemic countries: a geostatistical model-based map. PLoS Med.

[5] Howes RE, Battle KE, Satyagraha AW, Baird JK, Hay SI (2013). G6PD deficiency: global distribution, genetic variants and primaquine therapy. Adv Parasitol.

[6] Nkhoma ET, Poole C, Vannappagari V, Hall SA, Beutler E (2009). The global prevalence of glucose-6-phosphate dehydrogenase deficiency: a systematic review and meta-analysis. Blood Cells Mol Dis.

[7] Cappellini MD, Fiorelli G (2008). Glucose-6-phosphate dehydrogenase deficiency. Lancet.

[8] Luzzatto L, Mehta A, Vulliamy TJ, Scriver CR, Beaudet AL, Sly WS, Valle D (2001). Glucose-6-phosphate dehydrogenase deficiency. The Metabolic and Molecular Basis of Inherited Disease.

[9] Minucci A, Moradkhani K, Hwang MJ, Zuppi C, Giardina B, Capoluongo E (2012). Glucose-6-phosphate dehydrogenase (G6PD) mutations database: review of the “old” and update of the new mutations. Blood Cells Mol Dis.

[10] World Health Organization (1989). Glucose-6-phosphate dehydrogenase deficiency: WHO Working Group. Bull World Health Organ.

[11] World Health Organization (1967). Standardization of Procedures for the Study of Glucose-6-Phosphate Dehydrogenase: Report of a WHO Scientific Group. WHO Technical Report Series No. 366.

[12] Ainoon O, Alawiyah A, Yu YH, Cheong SK, Hamidah NH, Boo NY, Zaleha M (2003). Semiquantitative screening test for G6PD deficiency detects severe deficiency but misses a substantial proportion of partially-deficient females. Southeast Asian J Trop Med Public Health.

[13] Johnson MK, Clark TD, Njama-Meya D, Rosenthal PJ, Parikh S (2009). Impact of the method of G6PD deficiency assessment on genetic association studies of malaria susceptibility. PLoS ONE.

[14] Nantakomol D, Paul R, Palasuwan A, Day NP, White NJ, Imwong M (2013). Evaluation of the phenotypic test and genetic analysis in the detection of glucose-6-phosphate dehydrogenase deficiency. Malar J.

[15] Reclos GJ, Hatzidakis CJ, Schulpis KH (2000). Glucose-6-phosphate dehydrogenase deficiency neonatal screening: preliminary evidence that a high percentage of partially deficient female neonates are missed during routine screening. J Med Screen.

[16] Shah SS, Diakite SA, Traore K, Diakite M, Kwiatkowski DP, Rockett KA, Wellems TE, Fairhurst RM (2012). A novel cytofluorometric assay for the detection and quantification of glucose-6-phosphate dehydrogenase deficiency. Sci Rep.

[17] van Noorden CJ, Vogels IM, James J, Tas J (1982). A sensitive cytochemical staining method for glucose-6-phosphate dehydrogenase activity in individual erythrocytes. I. Optimalization of the staining procedure. Histochemistry.

[18] van Noorden CJ, Dolbeare F, Aten J (1989). Flow cytofluorometric analysis of enzyme reactions based on quenching of fluorescence by the final reaction product: detection of glucose-6-phosphate dehydrogenase deficiency in human erythrocytes. J Histochem Cytochem.

[19] Wei X, Ju X, Yi X, Zhu Q, Qu N, Liu T, Chen Y, Jiang H, Yang G, Zhen R, Lan Z, Qi M, Wang J, Yang Y, Chu Y, Li X, Guang Y, Huang J (2011). Identification of sequence variants in genetic disease-causing genes using targeted next-generation sequencing. PLoS ONE.

[20] Kim S, Nguon C, Guillard B, Duong S, Chy S, Sum S, Nhem S, Bouchier C, Tichit M, Christophel E, Taylor WRJ, Baird JK, Menard D (2011). Performance of the CareStart G6PD deficiency screening test, a point-of-care diagnostic for primaquine therapy screening. PLoS ONE.

[21] Kuwahata M, Wijesinghe R, Ho MF, Pelecanos A, Bobogare A, Landry L, Bugora H, Vallely A, McCarthy J (2010). Population screening for glucose-6-phosphate dehydrogenase deficiencies in Isabel Province, Solomon Islands, using a modified enzyme assay on filter paper dried bloodspots. Malar J.

[22] Tantular IS, Iwai K, Lin K, Basuki S, Horie T, Htay HH, Matsuoka H, Marwoto H, Wongsrichanalai C, Dachlan YP, Kojima S, Ishii A, Kawamoto F (1999). Field trials of a rapid test for G6PD deficiency in combination with a rapid diagnosis of malaria. Trop Med Int Health.

[23] Tinley KE, Loughlin AM, Jepson A, Barnett ED (2010). Evaluation of a rapid qualitative enzyme chromatographic test for glucose-6-phosphate dehydrogenase deficiency. Am J Trop Med Hyg.

[24] Kornberg A, Horecker BL, Colowick SP, Kaplan NO (1959). Glucose-6-phosphate dehydrogenase. Methods in Enzymology.

[25] Lohr GW, Waller HD, Bergmayer HU (1974). Glucose-6-phosphate dehydrogenase. Methods of Enzymatic Analysis.

[26] De Niz M, Othieno L, Mbabazi E, Nabukeera D, Ssemmondo E, Gonahasa S, Tumwebaze P, Diliberto D, Maiteki-Sebuguzi C, Staedke SG, Drakeley C (2013). Tools for mass screening of G6PD deficiency: validation of the WST8/1-methoxy-PMS enzymatic assay in Uganda. Malar J.

